# Biosynthesis and Response of Zinc Oxide Nanoparticles against Periimplantitis Triggering Pathogens

**DOI:** 10.3390/ma15093170

**Published:** 2022-04-27

**Authors:** Bernice Yii Shu Ting, Neeraj Kumar Fuloria, Vetriselvan Subrimanyan, Sakshi Bajaj, Suresh V. Chinni, Lebaka Veeranjaneya Reddy, Kathiresan V. Sathasivam, Sundram Karupiah, Rishabha Malviya, Dhanalekshmi Unnikrishnan Meenakshi, Neeraj Paliwal, Krishna Priya, Shivkanya Fuloria

**Affiliations:** 1 Faculty of Pharmacy, AIMST University, Bedong 08100, Kedah, Malaysia; berniceyst95@hotmail.com (B.Y.S.T.); sundram@aimst.edu.my (S.K.); neerajpaliwal@aimst.edu.my (N.P.); 2 Faculty of Medicine, Bioscience and Nursing, MAHSA University, Kuala Lumpur 42610, Selangor, Malaysia; drvetriselvan@mahsa.edu.my; 3 Delhi Institute of Pharmaceutical Science and Research (DIPSAR), Delhi Pharmaceutical Science and Research University (DPSRU), Pushp Vihar, New Delhi 110017, India; sakshibajaj84@gmail.com; 4 Department of Biotechnology, Faculty of Applied Sciences, AIMST University, Bedong 08100, Kedah, Malaysia; v_suresh@aimst.edu.my (S.V.C.); skathir@aimst.edu.my (K.V.S.); 5 Department of Microbiology, Yogi Vemana University, Kadapa 516005, India; lvereddy@yogivermanauniversity.ac.in; 6 Department of Pharmacy, School of Medical & Allied Sciences (SMAS), Galgotias University, Gautam Budha Nagar, Greater Noida 201310, India; rishabha.malviya@galgotiasuniversity.edu; 7 College of Pharmacy, National University of Science and Technology, Muscat 130, Oman; dhanalekshmi@nu.edu.om; 8 Manipal College of Dental Sciences, Manipal University, Mangalore 575001, India; krishna.priya3@learner.manipal.edu

**Keywords:** ZnPs, biosynthesis, microbiota, periimplantitis, stability, optimization

## Abstract

Periimplantitis due to pathogenic bacteria is considered as a major cause for dental implants failures. Biogenic zinc oxide nanoparticles (ZnPs) are known to inhibit periimplantitis triggering pathogens. The current investigation intended to perform ZnPs biosynthesis and evaluation against periimplantitis triggering bacteria. The current study involved ZnPs biosynthesis using *Andrographis paniculata* leaves aqueous extract (APLAE), followed by optimization, stability, characterization, and in vitro evaluation against periimplantitis triggering bacteria. The experimental results indicated the success of ZnPs biosynthesis based on the optimization of zinc acetate (1.5 g), plant extract (5 mL), pH 12, and temperature (25 °C), and using the stability study (absorbance between 365–370 nm) and characterization data exhibiting broad and shifted bands (in FTIR spectrum), the size was found to be below 98.61 nm (determined by FESEM and XRD spectra) and 71.54% zinc was observed in the EDX spectrum. Biogenic ZnPs exhibited a high inhibitory activity against periimplantitis-triggering pathogens (*E. coli* and *S. aureus*). Based on the experimental results, the present study concludes that biogenic ZnPs possess a high inhibitory potential against periimplantitis-triggering bacteria, and it is established that the biosynthesis of ZnPs using APLAE is a useful method.

## 1. Introduction

Dental implants (DIs) are under the increased risk for bacterial contamination during the whole period of the implant functioning in the oral cavity. Around 15 million new DIs are currently placed worldwide annually. The biological complications such as peri-implantitis (PI) are still a possible threat to DI success [1,2]. The human ecosystem comprises a 1:1 ration of bacteria and human cells [3]. A slight disturbance in this ratio may activate the bacterial pathogens that trigger periimplantitis. Evidence suggests triggering of periimplantitis by two important bacteria, namely *Staphylococcus aureus* (*S. aureus*) and *Escherichia coli* (*E. coli*) [4,5]. Continuous and long-term administration of conventional antibiotics against various infections lead to multiple drug resistance (MDR) and high mortality risk [6]. The proven use of metallic nanoparticles in various fields, such as dentistry, biomedical engineering, electronics, cosmetics, and medicines, have shown a marked increase in the study of metallic nanoparticles in last few decades [7,8,9]. ZnPs are well known for their impressively large band gaps, large binding energy, and high piezoelectric properties [10]. Evidence suggests biogenic ZnPs offer high antimicrobial properties [11,12], and enjoy the benefits of biosafety, low cost, nontoxicity, and biocompatibility [11,13]. The ability of biogenic ZnPs to inhibit pathogens that trigger periimplantitis always draws the attention of investigators of periimplantitis research [14,15]. Plant extract phytochemicals may act as good capping and stabilizing agents by assisting in the size control and distribution, and also in the stabilization of the surface of nanoparticles [16]. Hessien et al. also concluded in their study that neem extract acts as a capping agent when used in the stabilization of zinc oxide nanoparticles and nickel oxide nanoparticles [17]. Buono et al. found that biogenic ZnO nanoparticles help in the enhancement of physiological and biochemical traits in maize. Plants can gain benefits from biogenic metal oxide nanoparticles, and their synthesis avoids the environmental implications of traditional synthetic processes [18]. Ahmed et al. described that hazardous chemicals employed for NP synthesis subsequently become liable for numerous health problems due to their toxicity, posing severe environmental concerns, while other methods are costly and need a lot of energy for NP synthesis. However, for biological applications where purity is a priority, the biogenic synthesis process for producing NPs is environmentally friendly and free from chemical pollutants. Different biological entities, such as extracts, enzymes, or proteins from a natural product, are used in biological methods to minimize and stabilize the development of NPs. The structure, shape, size, and morphology of synthesized NPs are all influenced by the nature of these biological entities [19]. Hence, based on the problems of conventional antibiotics, periimplantitis, and triggering of periimplantitis by *S. aureus* and *E. coli*, as well as the benefits associated with biogenic ZnPs, the present study was designed to carry out ZnPs biosynthesis, optimization, characterization, and antimicrobial evaluation against periimplantitis triggering pathogens (*S. aureus* and *E. coli*).

## 2. Materials and Methods

### 2.1. Materials

The ZnPs were biosynthesized using *Andrographis paniculata* (*A. paniculata*) leaf aqueous extract (APLAE). The chemicals zinc acetate (ZnC_4_H_6_O_4_), sodium hydroxide (NaOH), and nutrient agar were procured from Fisher chemicals (Hampton, NH, USA), Sigma Aldrich (St. Louis, MI, USA), SD Fine (Mumbai, Maharashtra, India), and Hi-Media (Mumbai, Maharashtra, India). The glass wares were cleaned, washed with deionized water, and dried at 120 °C for 2 h. The plastic wares were autoclaved before initiation of the antimicrobial experiment.

### 2.2. APLAE Preparation

The experimental protocol for the preparation of APLAE was based on a standard procedure with slight modification [20]. Briefly, the fresh *A. paniculata* leaves were collected (from the premise of Butterworth, Penang, Malaysia), gently washed with water (to remove impurities), dried (40–50 °C), and powdered. The APLAE was prepared by boiling the mixture of *A. paniculata* leaf powder (25 g) in deionized water. The mixture was cooled and filtered using a four-fold muslin cloth and Whatman no. 1 filter paper to offer pure APLAE. Finally, the APLAE was stored at 8–10 °C in a refrigerator subjected to ZnPs synthesis.

### 2.3. ZnPs Biosynthesis

The ZnPs biosynthesis was performed as per the standard method, with minor modifications [13]. Briefly, to a mixture of 50 mL zinc acetate solution (prepared by mixing 1.5 g of zinc acetate into 50 mL deionized H_2_O) and 5 mL APLAE, 0.02 M NaOH solution was added to maintain the reaction mixture at pH 12. After 1 h of stirring, the prepared mixture, after being incubated overnight at room temperature, resulted in formation of white precipitates. The reaction conditions for the biosynthesis of ZnPs were based on the optimization of the zinc acetate concentration, APLAE volume, pH, and temperature. The precipitates were centrifuged several times by redispersion into deionized H_2_O, separated (by decantation), dried overnight, and preserved in airtight bottles for further studies. The ZnPs biosynthesis success was confirmed by UV–visible spectrometry. Small aliquots of biogenic ZnPs were diluted in deionized H_2_O (1 mL test sample with 4 mL deionized H_2_O). The test mixture obtained was subjected to UV–visible spectrometry analysis at room temperature to detect the surface plasmon resonance (SPR) peak. Absorbance was recorded between 400 to 800 nm using a Shimadzu U-2800 spectrophotometer (Shimadzu, Kyoto, Kyoto, Japan) running at a scanning speed of 300 nm/min.

### 2.4. Optimization of ZnPs Biosynthesis

The optimization of ZnPs biosynthesis was performed according to standard procedure, with minor modifications [13,18]. Briefly, various biosynthesis reaction mixtures of ZnPs were maintained with different parametric conditions, such as zinc acetate concentration (0.5, 1.0, 1.5, and 2.0 g), APLAE volume (2 mL, 4 mL, 5 mL, and 8 mL), pH (4, 7, 10, and 12), and temperature (4 °C, 25 °C, 60 °C, and 80 °C). All of the ZnPs biosynthesis reaction mixtures were subjected to visual examination (for change in color) and UV–visible spectrometry analysis (to observe SPR signal) after 24 h of incubation with the same reaction conditions, except for one parameter.

### 2.5. Stability of ZnPs

The optimized ZnPs biosynthetic mixture was subjected to a stability study for 2 h, 1 d, 3 d, 7 d, and 15 d. The ZnPs mixture was subjected to UV–visible spectrometry analysis for the SPR signal. The procedure for the stability study was conducted based on reported methods, with minor modifications [6].

### 2.6. Antimicrobial Activity of ZnPs

The biosynthesized ZnPs were tested for their inhibitory potential against periimplantitis triggering pathogens, namely *Staphylococcus aureus* (ATCC 29737) and *Escherichia coli* (ATCC 8739), using the well diffusion standard method with little modification [13,21]. Sawai et al. also used *Staphylococcus aureus* and *Escherichia coli* for the antifungal evaluation of metallic oxide powders like MgO, CaO, and ZnO [22]. Briefly, the fresh and pure culture of each bacterial strain was subcultured over nutrient broth maintained at 37 °C (previously shaken over rotary shaker maintained at 200 rpm). The strain of each bacterial culture was uniformly swabbed using sterilized cotton over an individual nutrient agar plate. Wells of 8 mm in width were drilled in the nutrient agar plates using gel puncture. In each well of the nutrient agar plate, ZnPs (1, 2, 4, 6, 8, and 10 mg/mL), APLAE (1, 2, 4, 6, 8 and 10 mg/mL), and ciprofloxacin (10 µg/mL) at a volume of 50 µL each were added using a micropipette. Lastly, the plates were subjected to incubation at 37 °C for 24 h, and the inhibited zones were measured in mm using a caliper.

### 2.7. Characterization of ZnPs

The ZnPs characterization was based on different techniques employed in various standard research [23,24,25]. Before initiation of the characterization study, ZnPs were purified to avoid the interaction of APLAE absorption in the ZnPs absorption spectrum. The ZnPs characterization involved recording the UV–visible spectrum in a range from 200–800 nm using a UV–visible spectrometer (Shimadzu U-2800, Kyoto, Japan); recording of IR spectrum using PerkinElmer SLE/MSC4/29 (Waltham, MA, USA); FESEM measurement to understand the morphology of ZnPs; XRD spectrum using X-ray diffractometer (Malvern panalytical, Malvern, UK) to dermine crystal nature; and EDX spectrum using FEI Nova Nano SEM 450 (Hillsboro, OR, USA) with EDX assembly.

## 3. Results

### 3.1. Biosynthesis of ZnPs

**Figure 1:**
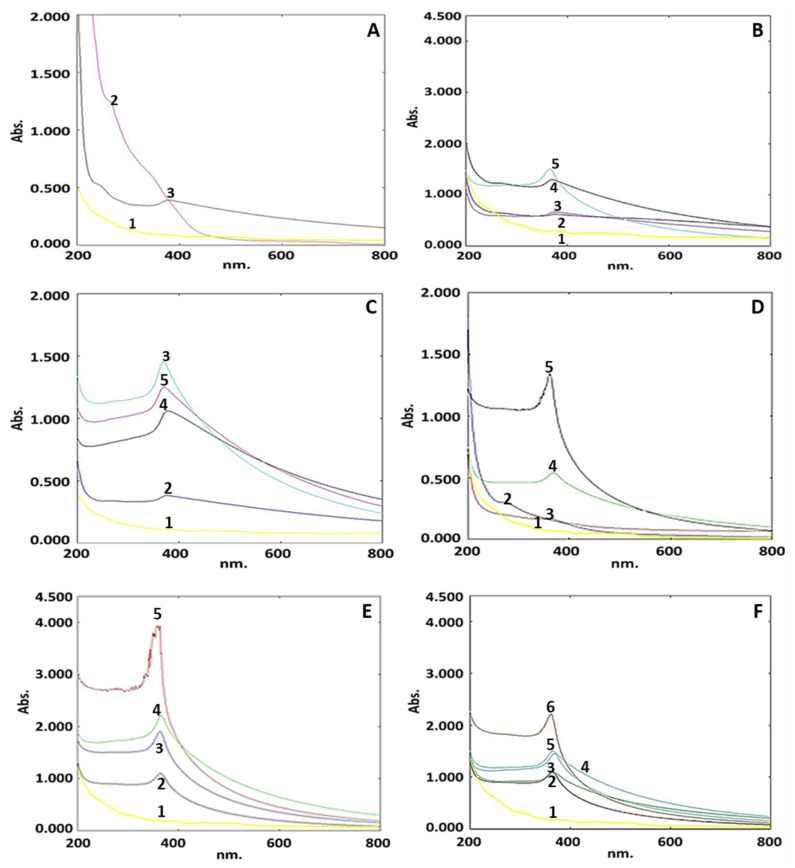
(**A**) UV–visible spectra for the ZnPs synthesis reaction mixture (with 1.5 g zinc acetate, 5 mL APLAE, pH 12, and at 25 °C) and pure APLAE corresponding to curves 2 and 3, respectively. (**B**) Optimization of ZnPs reaction mixture for different concentrations of zinc acetate solution (0.5, 1.0, 1.5, and 2.0 g corresponding to curves 2, 3, 4, and 5, respectively). (**C**) Optimization of ZnPs reaction mixture for different volumetric concentrations of APLAE (2, 4, 5, and 8 mL corresponding to curves 2, 3, 4, and 5, respectively). (**D**) Optimization of ZnPs reaction mixture for pH (4, 7, 8, and 12 corresponding to curves 2, 3, 4, and 5, respectively). (**E**) Optimization of ZnPs reaction mixture for temperature (4 °C, room temperature, 60 °C and 80 °C corresponding to curves 2, 3, 4, and 5). (**F**) Stability of ZnPs reaction mixture (for 2 h, 1 d, 5 d, 10 d, and 30 d corresponding to curves 2, 3, 4, 5, and 6). In all spectra, curve 1 represents the pure zinc acetate solution.

Formation of white precipitates in the incubated reaction mixture of zinc acetate and APLAE maintained at pH 12 was a preliminary confirmation for the ZnPs presence and biosynthesis. The UV–visible spectrometry analysis of pure APLAE and biogenic ZnPs offered an absorption spectrum shown in Figure 1A.

### 3.2. Optimization of ZnPs Biosynthesis Parameters

Optimization of ZnPs biosynthesis was based on the following four key parameters: zinc acetate, APLAE volume, pH, and temperature. The optimization of the results was validated based on the presence of UV–visible absorbance signal within the range claimed by other standard research [13].

#### 3.2.1. Optimization of Zinc Acetate Concentration

Biogenic ZnPs optimization based on four concentrations of zinc acetate (0.5, 1.0, 1.5, and 2.0 g) offered a UV–visible spectrum comprising four curves (curves 2, 3, 4, and 5), as shown in Figure 1B. Curve 5 shows signals between 355 and 362 nm, with a sharp signal at 362 nm. This demonstrates that 1.5 g of zinc acetate is the optimum concentration for ZnPs production. In curve 1 for the pure zinc acetate solution, the spectrum indicated no ZnP signal.

#### 3.2.2. Optimization of APLAE Volume

Biogenic ZnPs optimization based on four volumetric concentrations of APLAE (2 mL, 4 mL, 5 mL, and 8 mL) offered a UV–visible spectrum comprising four curves (curves 2, 3, 4 and 5), as shown in Figure 1C. The presence of the peak at 380 nm in curve 3 justified the selection of a 5 mL volume of APLAE as a requirement for ZnPs biosynthesis.

#### 3.2.3. Optimization of pH

Biogenic ZnPs optimization based on different pH (4, 7, 10 and 12) offered a UV–visible spectrum comprising four curves (curves 2, 3, 4, and 5), as shown in Figure 1D. Curve 5 shows a sharp signal at 385 nm, with signals ranging from 290 to 385 nm. This established pH 12 as the optimum condition for ZnPs production.

#### 3.2.4. Optimization of Temperature

Biogenic ZnPs optimization based on different temperatures (4 °C, room temperature, 60 °C, and 80 °C) offered a UV–visible spectrum comprising four curves (curves 2, 3, 4, and 5), as shown in Figure 1E. There was no sharp absorption signal in curve 5 (associated to 80 °C), but three signals for ZnPs at 369, 373, and 373 nm in curves 2, 3, and 4 (related to 4 °C, room temperature, and 60 °C) were observed. This reinforced the assumption that room temperature (curve 3) was the best temperature for the biosynthesis of ZnPs.

### 3.3. Stability of ZnPs

The ZnPs subjected to the UV–visible analysis-based stability study (for 2 h, 1 d, 5 d, 10 d, and 30 d) offered a UV–visible spectrum comprising five curves (curves 2, 3, 4, 5, and 6), as shown in Figure 1F.

### 3.4. Characterization of ZnPs

#### 3.4.1. Fourier Transformed Infrared (FTIR) Analysis

**Figure 2:**
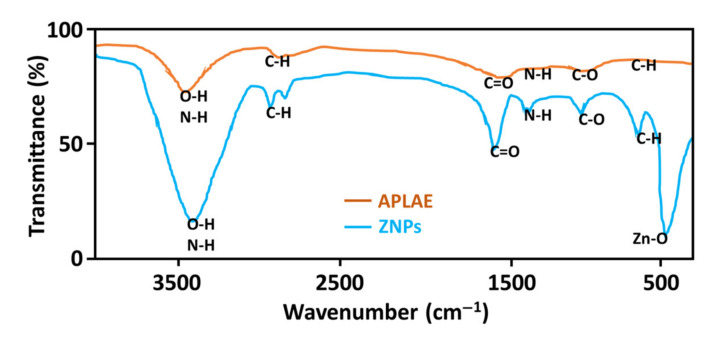
FTIR spectrum of pure APLAE and biosynthesized ZnPs.

The FT-IR analysis of the APLAE and ZnPs offered a spectrum with two curves, as given in Figure 2. The APLAE curve displayed characteristic narrow vibrational bands at 3467 cm^−1^ (O–H vibrations), 2821 cm^−1^ (C–H vibrations), and 1616 cm^−1^ (C=O vibrations). The ZnPs curve displayed shifted broad bands at 3469 cm^−1^ (O–H vibrations), 2904 cm^−1^ (C–H vibrations), 1668 cm^−1^ (C=O vibrations), and 452 cm^−1^ (Zn–O). The FTIR data were supported in the standard literature [26].

#### 3.4.2. Field Emission Scanning Electron Microscopy (FESEM)

**Figure 3:**
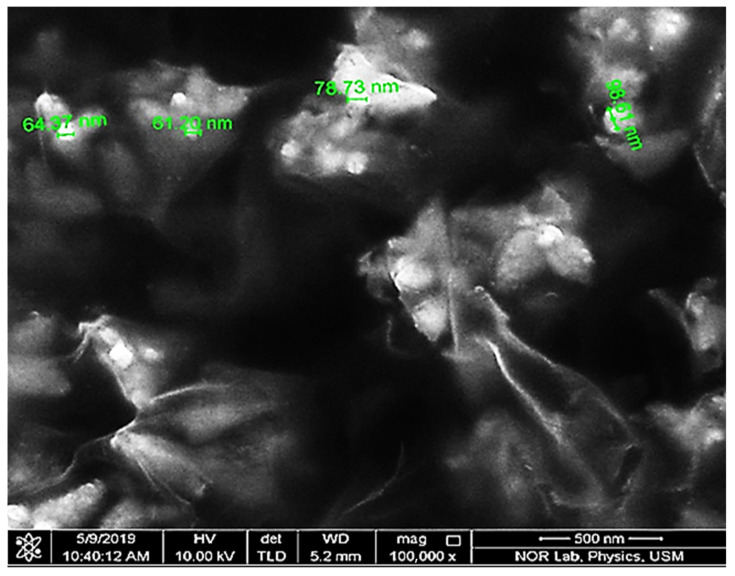
ZnPs FESEM image.

FESEM was used to investigate the morphology of biosynthetic ZnPs, and offered an FESEM image of ZnPs given in Figure 3. The FESEM study determined the biosynthesized ZnPs to possess a size below 98.61 nm. The ZnPs particles size determined through FESEM was found to be in agreement with the size calculated using the Debye–Scherrer equation through the XRD study.

#### 3.4.3. X-ray Diffraction (XRD) Analysis

**Figure 4:**
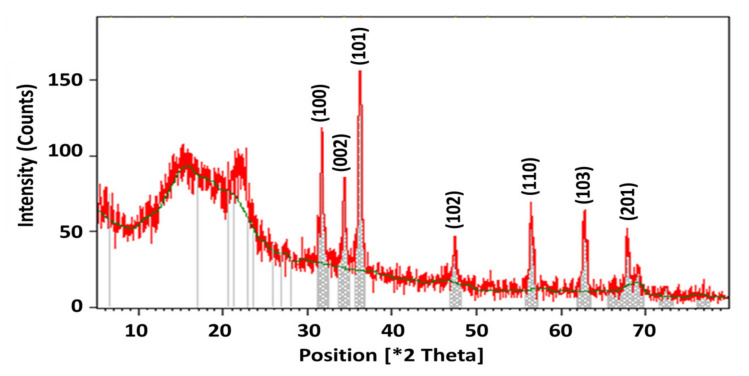
ZnPs XRD image.

The application of XRD analysis, used to understand the crystal nature of ZnPs, offered the XRD spectrum given in Figure 4. The spectrum exhibited distinctive diffraction peaks at 2θ values of 31.70°, 34.33°, 36.19°, 47.45°, 56.52°, 62.78°, and 69.02°, indexed to 100, 002, 101, 102, 110, 103, and 201 planes, respectively.

The size of the ZnPs was calculated using the Debye–Scherrer formula given in Equation (1).

(1) $$D=\frac{K\lambda }{\beta \mathrm{Cos}\theta }$$

where D is the particle size in nm, λ is the X-ray wavelength, θ is the Braggs’ angle of reflection, and β is the full width at half maximum (FWHM). The Debye–Scherrer formula-based data are given in Table 1.

#### 3.4.4. Energy Dispersive X-ray Diffraction (EDX) Analysis

**Figure 5:**
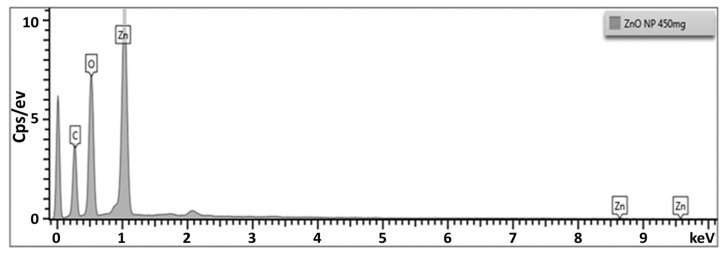
ZnPs EDX spectrum.

The EDX analysis was used to understand the presence of the elemental constitution of ZnPs, and offered the EDX spectrum given in Figure 5. The EDX spectrum revealed a strong signal in the zinc region (wt. 71.54%) and oxygen region (24.2%), which confirmed ZnPs formation. The existence of a trace quantity of carbon indicates that APLAE biomolecules were involved in the reduction and capping of biogenic ZnPs [27].

### 3.5. Antimicrobial Activity of ZnPs

The extensive use of ZnPs as an antimicrobial suggests their potential in combating periimplantitis-triggering microbes [14,15]. Plant-extract-blended ZnPs have a high potential to inhibit periimplantitis-causing microorganisms [28]. In thecurrent investigation, biogenic ZnPs were tested for their response against periimplantitis-triggering bacteria, namely *E. coli*, and *S. aureus*, using the well diffusion method (data are given in Table 2).

## 4. Discussion

In the present investigation, ZnPs were biosynthesized by utilizing the aqueous extract of *A. paniculate* leaves. The leaves of this herb are known to contain phenolic compounds, including flavonoids, alkaloids, and tannins [29], which enables it to coat and complex with zinc cations and results in the white powder of stabilized ZnPs. The ZnPs synthesis reaction progress was monitored using a UV–visible spectrometer. Figure 1A exhibits a characteristic signal 379 nm in curve 3 for ZnPs, attributed to their large excitation binding energy, whereas curve 2 exhibits no signal for pure APLAE. The spectrum revealed no ZnP signal in curve 1 for the pure zinc acetate solution. The evidence suggests that the band gap increases with a decrease in the particle size, and is inversely related to the wavelength of absorption [30]. The research claims that ZnO absorption occurs near 385 nm, supporting the absorption of biogenic ZnPs at 379 nm. The shift in the absorbance of ZnPs could be attributed to a decrease in the particle size.

In order to have the best yield of ZnPs, the reaction mixture for the biosynthesis of ZnPs was optimized based on the standard literature [31,32]. The UV–visible spectrometry-based optimization study identified the key parameters for the synthesis of ZnPs using APLAE, namely: concentration of zinc acetate, volumetric ratio of concentration of APLAE to zinc acetate, pH, and temperature for the green synthesis of ZnPs. Optimization of ZnPs biosynthesis was based on five curves, namely 1, 2, 3, 4, and 5, shown in Figure 1B, that exhibited signals between 355–362 nm, with a sharp signal at 362 nm in curve 5. This confirms 1.5 g as the optimum concentration of zinc acetate for ZnPs biosynthesis. The spectrum revealed no ZnP signal in curve 1 for the pure zinc acetate solution. The increase in zinc acetate concentration from 0.5 g to 2 g caused the immediate initiation of the bio-reduction process, which led to the biosynthesis of ZnPs with the average size ranging from 61.20 nm–98.61 nm due to large anisotropic particle formation [31]. Among the four concentrations, 1.5 g was selected, as at this concentration, a sharp signal of ZnPs appeared. The optimization of the green synthesis of ZnPs based on four volumetric concentrations APLAE (2 mL, 4 mL, 5 mL, and 8 mL) under UV–visible spectrometry offered a UV–visible spectrum, given in Figure 1C, that comprised four curves, namely 2, 3, 4, and 5, exhibiting signals between 372–380 nm. The spectrum revealed no ZnP signal in curve 1 for the pure zinc acetate solution. The presence of a peak at 380 nm in curve 3 justifies the selection of 5 mL volume of APLAE as a requirement for ZnPs biosynthesis [31]. The optimization of the green synthesis of ZnPs based on different pH (4, 7, 8, and 12) under UV–visible spectrometry analysis offered the spectrum given in Figure 1D, that comprised four curves, namely 2, 3, 4, and 5, exhibiting signals between 290–385 nm, with a sharp signal at 385 nm in curve 5. The spectrum revealed no ZnPs signal in curve 1 for the pure zinc acetate solution. This confirmed pH 12 as the optimum requirement for ZnPs biosynthesis. A change in pH that causes a change in the electrical charges of biomolecules could change the reduction and capping property of biomolecules and later the growth of ZnPs [31]. Optimization of the biosynthesis of ZnPs based on different temperatures (4 °C, room temperature, 60 °C, and 80 °C) under UV–visible spectrometry offered the UV–visible spectrum given in Figure 1E, that exhibited no sharp absorption signal in curve 5 (related to 80 °C), whereas there were three signals for ZnPs in curves 2, 3, and 4 (related to 4 °C, room temperature, and 60 °C) at 369, 373, and 373 nm, respectively. This supported room temperature (representing curve 3) as the ideal condition to biosynthesise ZnPs.

The stability study of ZnPs (for 2 h, 1 d, 5 d, 10 d, and 30 d) showed a UV–visible spectrum comprising five curves, namely 2, 3, 4, 5, and 6, given in Figure 1F, exhibiting signals in the range of 365–370 nm. The spectrum illustrated an increase in ZnPs absorbance with time and represented ZnPs stability until 30 days based on the retention of the ZnPs signal in the range of 365–370 nm. The spectrum revealed no ZnP signal in curve 1 of the pure zinc acetate solution. The present study ZnPs signal range has also been supported by other research studies [6,32].

After successful optimization and stability studies, the biosynthesized ZnPs were further subjected to characterization studies, such as FTIR, FESEM, XRD, and EDX analysis, to determine the morphology and structure of ZnPs. Prior to the characterization studies, the biogenic ZnPs were repeatedly washed and centrifuged using deionized water. This was done to avoid any chance of unbound residual biochemical moieties of APLAE interfering with the FTIR, FESEM, XRD, and EDX data of ZnPs. The shifting and broadening of bands in the ZnPs FTIR spectrum were attributed to the oxidation and reduction of zinc acetate to zinc oxide by the phytochemical moieties present in the APLAE and formation of ZnPs. The appearance of a peak at 452 cm^−1^ in the ZnPs FTIR spectrum, corresponding to the stretching of the ZnO bond, also confirmed the formation of ZnPs. Based on a comparison of APLAE and the biosynthesized ZnPs FTIR spectra, the FTIR study recognized APLAE as exhibiting dual capping (stabilizing), as well as an oxidizing and reducing property [33].

The biogenic ZnPs were subjected to FESEM analysis to establish the size, shape, and distribution of green ZnPs. The FESEM micrograph given in Figure 3 determined the diverse magnifications and confirmed that biosynthesized ZnPs had a petal shaped structure attached together to form a flower-like morphology with irregular shapes. The FESEM analysis revealed that the size of ZnPs ranged between 61.20 nm and 98.61 nm. The results of this study were also supported by other standard research [34].

XRD spectrometry assisted in the determination of the crystal nature of biosynthetic ZnPs and was also supported by other research. The crystalline nature of ZnPs was confirmed by analysis of the XRD pattern (Figure 4). No characteristic peaks of any impurities were detected, suggesting that high quality ZnPs were produced. The average crystallite size of the ZnPs was found to be less than 100 nm, and was calculated using the Debye–Scherrer formula [6].

The EDX analysis, which aimed to understand the presence of the elemental constitution of ZnPs, produced the EDX spectrum given in Figure 5. The EDX spectrum revealed a strong signal in the zinc region (wt. 71.54%) and oxygen region (24.2%), which confirmed the formation of ZnPs. The existence of a trace quantity of carbon indicates that APLAE biomolecules are involved in the reduction and capping of biogenic ZnPs [27].

The antimicrobial data given in Table 1 revealed that the inhibition zone of biogenic ZnPs was much higher than for pure APLAE. The biogenic ZnPs exhibited a maximum zone of inhibition against *S. aureus* (25.0 mm at 10 mg/mL) and a lesser zone of inhibition against *E. coli* (22.0 at 10 mg/mL). In comparison to ZnPs, pure APLAE exhibited a lesser zone of inhibition of against *E. coli* (16 mm at 10 mg/mL) and no zone of inhibition against *S. aureus*. Interestingly, a pattern was observed in the antimicrobial activity of newer ZnPs; when the concentration of ZnPs increased from 1 mg/mL to 10 mg/mL, there was a significant increase in the zone of inhibition. This pattern of increment in antimicrobial response due to the biochemical moieties of the plant extract (used for the biosynthesis of ZnPs) was also supported by other investigations [35]. The antimicrobial activity results indicated that capping of zinc with biochemical moieties of APLAE caused a marked increase in the antimicrobial potential of ZnPs. This is based on the evidence that *A. paniculata* leaves to possess terpenes, tannins, tocopherols, flavonoids, and alkaloids [36]. The experimental results recognize the high antibacterial potential of ZnPs in the treatment of periimplantitis (formulated using APLAE) triggered by *E. coli* and *S.aureus*. The antimicrobial results of ZnPs (formulated using APLAE) were comparable to a conventional antibiotic (ciprofloxacin). Based on the pattern of antimicrobial response offered by ZnPs against periimplantitis-triggering bacteria in the present study, it can be inferred that small-sized ZnPs (biosynthesized from APLAE), when increased in concentration from a lower dose (1 mg/mL) to a higher dose (10 mg/mL), leads to an increase in antimicrobial response against periimplantitis-triggering bacteria (*E. coli* and *S. aureus*). The fact that ZnPs that are smaller in size and higher in dose exhibit a higher antimicrobial potential was also supported by other investigations [25]. The zone of inhibition-based evaluation of the antimicrobial activity of zinc oxide nanoparticles with plant extract and a broad-spectrum antibiotic (like ciprofloxacin) in the present study was also supported by several other studies [37,38,39,40,41].

The nanoparticles toxicity is connected to damage of the bacterial cell membrane, resulting in ZnPs entry into and to their accumulation in the cytoplasm [42]. The impact on bacterial growth depends on the nanoparticles’ concentration, shape, size, agglomeration, and media pH [43,44].

Evidence suggests metal oxide nanoparticles damage the bacteria via diffusion through the bacterial cell membrane and DNA. Evidence suggests ZnPs in presence of moisture produces reactive oxygen species (superoxides, hydrogen peroxide, and hydroxyl radicals) that apparently react with bacterial cell biomolecules (like protein, DNA, and lipids), and eventually causes cell apoptosis [45,46]. The in-vitro and in-vivo studies of Yan et al. and Fan et al. also provided evidence for ZnPs’ potential to induce cell apoptosis [47,48]. The exact mechanism of cytotoxicity of ZnPs is still under debate, although several proposed mechanisms have been suggested and adopted. The fundamental mechanism of ZnPs cytotoxicity involves the intracellular release of zinc ions, supplemented by the induction of ROS [49]. Zinc oxide exhibits an amphoteric property, as it reacts with both acid and alkali, leading to Zn^2+^ ions [46]. As per the antimicrobial results of the present study and other literary evidence, it can be postulated that biochemical moieties of APLAE cause a capping of zinc oxide and lead to a marked increase in the antimicrobial potential of ZnPs against periimplantitis-triggering bacteria. The present study is a preliminary work on the biosynthesis of ZnPs from *A. paniculata* leaf extract. The study revealed that ZnPs obtained from *A. paniculata* leaves exhibited a strong response against *S. aureus* and *E. coli*. Previously, several studies determined a biogenic ZnPs antimicrobial potential against *S. aureus* and *E. coli* [10,50]. However, this is the first-time that the antimicrobial potential of ZnPs obtained from *A. paniculata* leaf aqueous extract has been related to periimplantitis. Apart from, in Malaysia, this is the first-time study wherein ZnPs are biosynthesized using *A. paniculata*. In the future, this method could be employed in newer drug delivery systems to prevent periimplantitis, because of its cost effectiveness in comparison to other conventional approaches.

## 5. Conclusions

The visual examination of white precipitate formation, as well as UV–visible spectrometry and FTIR spectrometric data of the present study confirmed the success of the biosynthesis of zinc oxide nanoparticles using APLAE. The FESEM, EDX, and XRD data established the morphology of ZnPs with a size smaller than 98.61 nm, and showed them to be well dispersed and have a petal shaped structure that attached together to form a flower-like morphology with an irregular shape. The antimicrobial activity of ZnPs against periimplantitis-triggering bacteria formulated in the present study established that small-sized ZnPs (biosynthesized from APLAE), when increased in concentration from a lower dose to a higher dose, leads to an increase in antimicrobial response. Hence, the present study concludes that zinc oxide nanoparticles obtained from APLAE exhibit a strong response against *S. aureus* and *E. coli*, and recommends APLAE as a potential source for the green production of potent zinc oxide nanoparticles for the prevention of periimplantitis.

## Figures and Tables

**Table 1:** XRD-based parametric calculation for the determination of the average particle size of the ZnPs.

2θ	hkl	FWHM (β)	D (nm)
31.70	100	0.9890	87.18
34.33	002	0.9700	88.89
36.17	101	0.9971	86.17
47.45	102	0.9163	98.14
56.52	110	0.9515	98.97
62.78	103	0.9859	98.56
69.02	201	0.9850	98.65

**Table 2:** Zone of inhibition (expressed in mm ± standard deviation).

Microorganism	Zone of Inhibition in mm												
	ZnPs						APLAE						Ciprofloxacin
Concentration (mg/mL)	1.0	2.0	4.0	6.0	8.0	10.0	1.0	2.0	4.0	6.0	8.0	10.0	10 µg/mL
*S. aureus*	16 ± 1	18 ± 1	18.5 ± 0.5	19 ± 2	19 ± 3	25 ± 1	N/A	N/A	N/A	N/A	N/A	N/A	21 ± 0.58
*E. coli*	8 ± 1	10 ± 0.57	15 ± 0.57	19 ± 1	20.5 ± 1.5	22 ± 0.57	N/A	N/A	N/A	N/A	15.5 ± 1.5	16 ± 0.57	20 ± 0.00

## Data Availability

Data are contained within the article.
